# Structured evaluation of a comprehensive microsurgical training program

**DOI:** 10.6061/clinics/2021/e3194

**Published:** 2021-10-05

**Authors:** Tiago Guedes da Motta Mattar, Gustavo Bispo dos Santos, João Paulo Mota Telles, Marcelo Rosa de Rezende, Teng Hsiang Wei, Rames Mattar

**Affiliations:** I Divisao de Cirurgia da Mao e Microcirurgia Reconstrutiva, Instituto de Ortopedia e Traumatologia (IOT), Hospital das Clinicas HCFMUSP, Faculdade de Medicina, Universidade de Sao Paulo, Sao Paulo, Sao Paulo, SP, BR.; II Faculdade de Medicina FMUSP, Universidade de Sao Paulo, Sao Paulo, SP, BR.

**Keywords:** Microsurgery, Curriculum, Reconstructive Surgical Procedures, Education, Medical

## Abstract

**OBJECTIVES:**

This study proposed a structured microsurgical training program and evaluated it with the assistance of a large sample of surgeons.

**METHODS:**

The practical course comprised 16 sessions of approximately 4 hours each. This included two sessions for suturing rubber gloves and two sessions for suturing arteries, veins, and nerves in chicken thighs. The other sessions were performed on the femoral vessels of rats: 5 sessions for end-to-end arterial anastomosis, 5 for end-to-end venous anastomosis, 1 for arterial grafting, and 1 for end-to-side anastomosis. We conducted a structured assessment of the microsurgical skills in each training session.

**RESULTS:**

In this study, 89 surgeons were evaluated. The mean scores for the different procedures were as follows: glove suturing, 33.3±0.59; chicken nerve end-to-end anastomosis, 40.3±0.49; chicken artery suturing, 40.9±0.36; chicken vein suturing, 42.3±0.36; graft interposition, 44.8±0.7; and end-to-side anastomosis, 43.7±0.63 (*p*<0.05 for all). The chicken thigh suturing scores were significantly higher than the rubber gloves suturing scores (*p*<0.01). There were no differences between scores of the rat artery and chicken thigh suturing procedures (*p*=0.24). The rat venous anastomosis scores were higher than the rat arterial anastomosis scores (*p*=0.02), as were graft interposition scores when compared with end-to-end venous anastomosis scores. The end-to-side anastomosis scores did not differ significantly from the grafting scores (*p*=0.85). The most common errors were inadequate knotting technique and suture rupture due to inadequate technique (both n=88 [98.9%]).

**CONCLUSION:**

We propose a 16-step, progressive microsurgical training program to learn the basic microsurgical techniques comprehensively and reliably. The program was evaluated in a large sample of trainees, and it demonstrated the adequacy of the training sequence and results.

## INTRODUCTION

Microsurgery is paramount for performing complex reconstructive surgery and is an essential technical skill in many surgical areas. Many different standardized microsurgical training programs exist (1-3), which should ideally minimize the variations in surgical results. However, to assess microsurgical skills, one needs an objective, cost-effective, and reliable evaluation method (4,5).

Neuropsychological learning models show that there are substantially different stages in acquiring new knowledge, particularly regarding motor tasks and skills (6,7). The initial phases are characterized by fast improvement; subsequently, one reaches a plateau, in which there is marginal improvement and task automatization (6,7). Regarding microvascular anastomosis, Mokhtari et al. (8) demonstrated the existence of plateaus through the 24 microanastomosis sessions using tubes of progressively lower caliber.

Although many proposed microsurgical curricula exist (9-12), their evaluations are frequently performed in small samples of trainees (1,13-15). This study proposed a structured microsurgical training program and evaluated it with the assistance of a large sample of surgeons trained at a reference center.

## METHODS

### Program and participants

The Microsurgery Laboratory of our institution has a standard microsurgical training program composed of a theoretical introduction and 16 sessions of practical training. The students are exposed to introductory lectures regarding the operative microscope, instruments, and basic techniques for sutures and microanastomoses.

The practical course comprises 16 sessions of approximately 4 hours each. This includes two sessions for suturing rubber gloves and two for arterial, venous, and nerve suturing in chicken thighs. The other sessions are performed on rat femoral vessels: 5 sessions for end-to-end arterial anastomosis, 5 for end-to-end venous anastomosis, 1 for arterial grafting, and 1 for end-to-side anastomosis. Table 1 summarizes the training steps.

In total, 89 participants were evaluated: 13 hand surgery residents and 76 surgeons from other surgical backgrounds. The exclusion criteria were abandoning the program and refusal to assess their skills.

### Evaluation tool

We applied a previously validated tool, the Structured Assessment of Microsurgery Skills (SAMS) (15), to each training session. The SAMS tool comprises three major components: Global Rating Score (GRS), errors, and summative rating.

The GRS is composed of 12 items that evaluate 4 axes: dexterity, visuospatial ability, operative flow, and judgment. In the dexterity component, steadiness and handling of instruments and tissues are assessed. Dissection, knot technique, and suture placement are evaluated in the visuospatial component. The operative flow is evaluated based on the steps, motion, and speed. Finally, judgment is evaluated based on irrigation, patency test, and bleeding control. Each item is scored on a scale of 1 to 5, wherein higher grades represent better performance.

The descriptive list of errors is indicative of the typical mistakes made in the four ability axes and errors made during surgical planning. The overall performance is graded on a scale of 1 to 5 to provide summarized feedback to the student. In this study, a single experienced instructor evaluated all participants.

### Statistical analysis

Quantitative data were evaluated using the Shapiro-Wilk normality test and expressed as means (standard deviations) or medians (interquartile ranges), as appropriate. To evaluate improvement across training steps, a repeated-measures analysis of variance was used. Post-hoc paired comparisons between training steps were performed using Tukey’s method and Bonferroni correction. Qualitative data are described as frequencies (valid percentages) and were compared using the chi-squared test. All analyses were performed using IBM Statistical Product and Service Solutions Statistics for Windows, version 23.0. Ethical appraisal was provided by the IOT-FMUSP’s local Institutional Review Board (Protocol number: 1116).

## RESULTS

**Figure 1:**
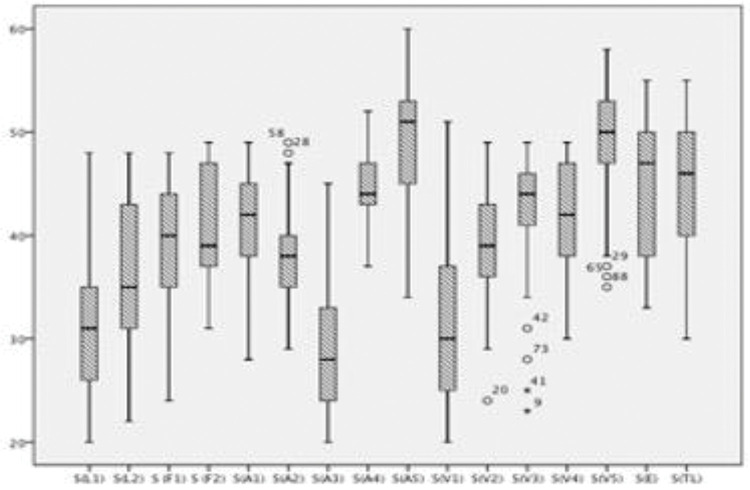
Scores in each training session. The box plots show the Structured Assessment of Microsurgery Skills scores of each session. S, session; L, rubber glove; F, chicken thigh; A, rat femoral artery end-to-end anastomosis; V, rat femoral vein end-to-end anastomosis; E, graft; TL, end-to-side anastomosis.

**Figure 2:**
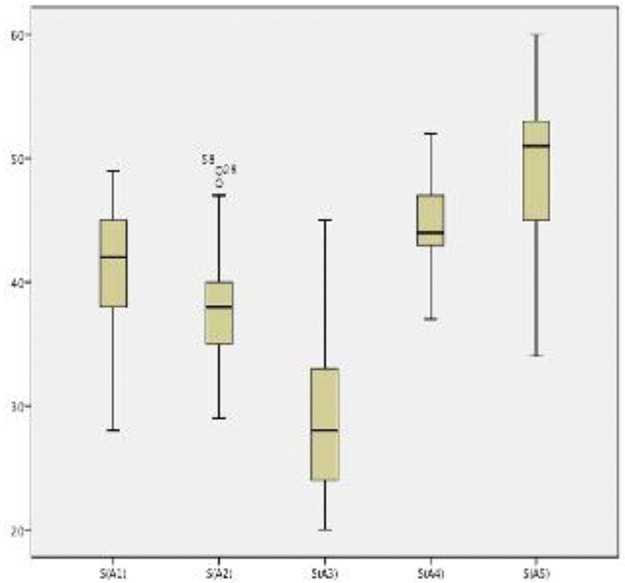
Scores in end-to-end arterial anastomosis sessions. The box plots show the distribution of scores in end-to-end arterial anastomosis sessions. A gradual increase was observed (*p*<0.05).

**Figure 3:**
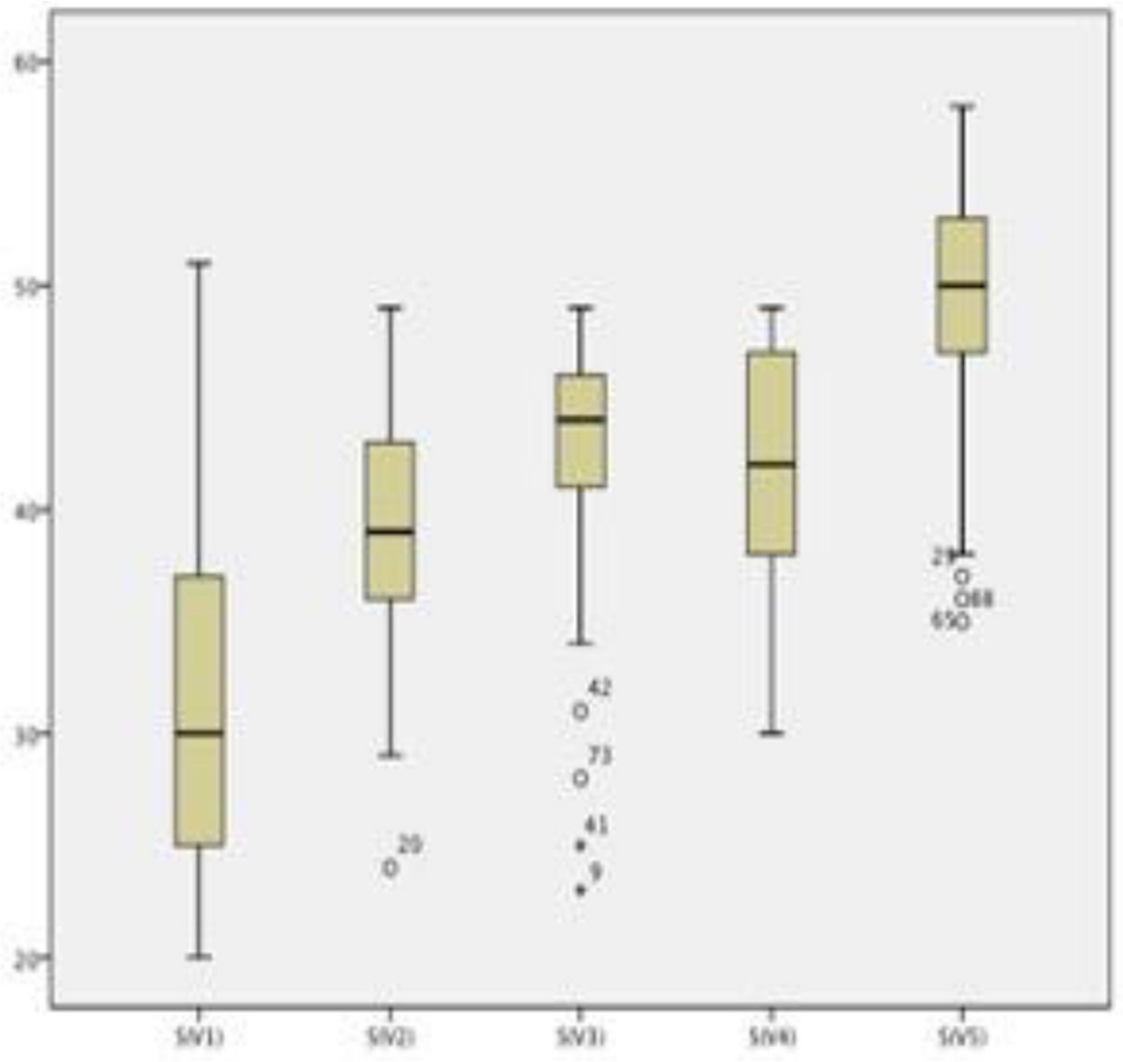
Scores in end-to-end venous anastomosis sessions. The box plots show the distribution of scores in end-to-end venous anastomosis sessions. A gradual increase was observed (*p*<0.05).

Boxplots showing the scores of each of the 16 training sessions are shown in Figure 1. The scores of both end-to-end arterial anastomosis (Figure 2) and end-to-end venous anastomosis (Figure 3) showed an increasing trend (*p*<0.01 for both), and their median scores surpassed 50 in the fifth session.

**Figure 4:**
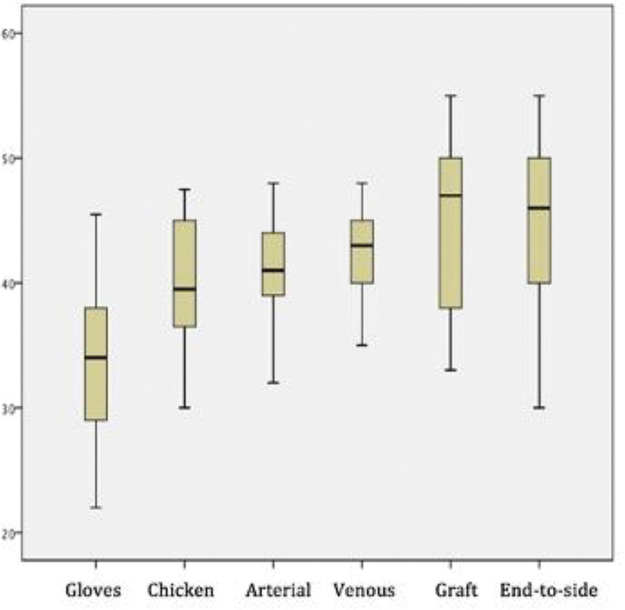
Scores in different skills. The box plots show pooled scores of different skills (*p*<0.05).

Figure 4 depicts the scores of each sequential step of the training program. The mean scores of the different procedures were as follows: glove suturing, 33.3±0.59; chicken nerve end-to-end anastomosis, 40.3±0.49; chicken artery suturing, 40.9±0.36; chicken vein suturing, 42.3±0.36; graft interposition, 44.8±0.7; and end-to-side anastomosis, 43.7±0.63 (*p*<0.05 for all).

**Figure 5:**
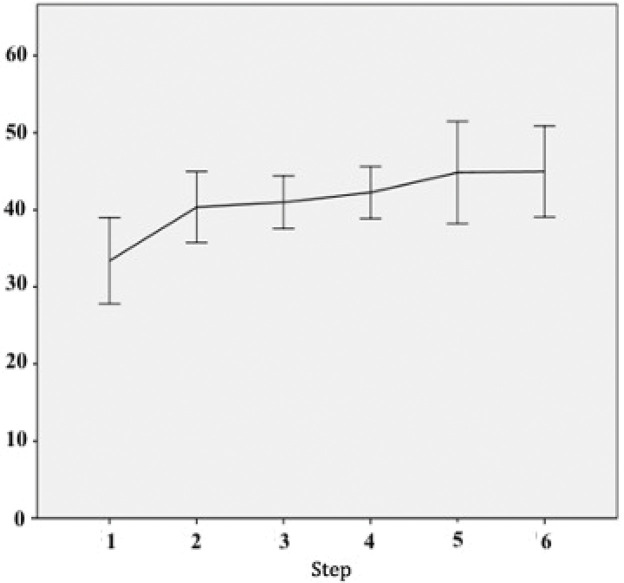
Learning curve across tasks. Despite the increasing difficulty of the tasks, the increasing scores (*p*<0.05) indicate that this is an adequate training sequence.

The chicken thigh suturing scores were significantly higher than the rubber gloves suturing scores (*p*<0.01). There were no significant differences between scores of the rat artery and chicken thigh suturing procedures (*p*=0.24). The rat venous anastomosis scores were higher than the rat arterial anastomosis scores (*p*=0.02), as were graft interposition scores when compared with end-to-end venous anastomosis scores. Moreover, the end-to-side anastomosis scores did not differ significantly from the grafting scores (*p*=0.85). Figure 5 shows a learning curve across the sequential steps, demonstrating progressively increasing scores.

Skills of the hand surgery residents were compared with those of other participants in each step of the training; there were no significant differences between the groups (*p*=0.11-0.37).

### Errors

The list of errors and number (percentage) of participants committing each type of error are shown in Table 2. Errors A-D refer to surgical planning, E-J to dexterity, K-P to visuospatial abilities, Q-S to operational errors, and U-Z to judgment. The most common errors were inadequate knotting technique and suture rupture due to inadequate technique (both n=88 [98.9%]).

## DISCUSSION

The GRS is a reliable and validated tool for analyzing the performance in surgical procedures (16,17). A structured and objective learning evaluation can improve performance in the operating room (18). The SAMS tool is being increasingly used to evaluate microsurgical skills. We believe our program is more interesting and unique than other programs, as it not only provides a sequential application of different materials but also minimizes the use of live animals while still exposing the trainee to high-fidelity scenarios (19-22). The advantages of a detailed structured evaluation include identifying specific strengths and weaknesses to improve the student’s techniques rather than only observing the outcomes (23,24).

In a study by Chan et al. (15) describing the SAMS evaluation for microsurgical anastomosis, the trained consultant and trainees had a mean GRS score of 54 (±3.2) and 37.6 (±4.7), respectively. The trainees in our study showed a progressively increasing score, reaching an average of approximately 45 points in graft training and 44 points in end-to-side anastomosis. Therefore, we believe that this training program provides adequate training for all essential microsurgical anastomosis techniques. Our sample size is also significant, comparable to or even higher than that in most of the published literature (1,13,15,20,24).

### Progressive skills and techniques

Sequential performance assessments provide interesting insights. In both arterial and venous end-to-end anastomoses, one can observe a clear improvement trend within the groups and a natural decrease in performance when the next step is initiated, as expected. The transition from suturing rubber gloves to suturing chicken thighs was accompanied by a significant increase in score, even though the latter task was much more complicated. We believe this underscores the importance of first contact with the microscope and suture lines before attempting to anastomose a vessel.

The transition from chicken thighs to rat femoral arteries did not show significant improvement (*p*=0.24), but the absolute mean score increased despite the significantly more complex scenario of the living model. We believe that this establishes the chicken thigh model as a reliable and efficient preparatory step for live anastomoses. The next steps, *i.e.*, end-to-end venous anastomosis and arterial graft interposition, had significantly higher scores than those of the previous step, despite being more technically challenging.

The end-to-side anastomosis is a different skill from all the former steps, which only involved end-to-end suturing. That is, our interpretation of the absolute score decreased, albeit statistically insignificant (*p*=0.85).

### Future directions

Surgical training is an infinite source of debate (25,26). The requirement of live animals for surgical training is of particular interest for many researchers (27-29). Nevertheless, most surgeons consider that adequate microsurgical training can be completed without live models, and the current effort is to attempt to minimize the use of live animals (30,31). The use of virtual and augmented reality devices will hopefully be a powerful adjunct to microsurgical training, but these tools still require a thorough validation (32).

## CONCLUSION

We propose this 16-step, progressive, microsurgical training program that combines multiple models to learn the basic microsurgical techniques comprehensively and reliably. The program was evaluated in a large sample of trainees, and it demonstrated the adequacy of the training sequence and results.

## AUTHOR CONTRIBUTIONS

Mattar TGM was responsible for the study conception, data acquisition, methodology and manuscript writing. Santos GB was responsible for the study conception, data acquisition and manuscript writing. Telles JPM was responsible for the data analysis, methodology and manuscript writing. Rezende MR was responsible for the study conception, data acquisition and supervision. Wei TH and Mattar Júnior R were responsible for the supervision, coordination and writing the final version of the manuscript.

## Figures and Tables

**Table 1:** Training program particulars.

Step	Number of sessions
Rubber glove suturing: progressively thinner suture lines (starting with 7-0 and ending with 10-0)	2
Chicken thigh training: arterial, venous, and nerve end-to-end anastomoses	2
End-to-end anastomosis in live rat femoral arteries	5
End-to-end anastomosis in live rat femoral veins	5
Arterial graft interposition in live rat femoral arteries	1
End-to-side anastomosis from rat femoral artery to vein	1

**Table 2:** List of errors.

Error	N (%)
A - Inadequate operative field	46 (51.7)
B - Inadequate vessel preparation	0
C - Lost focus	70 (78.7)
D - Loss of central vision	73 (82)
E - Reapplying clamp	72 (80.9)
F - Broken needle/suture	40 (44.9)
G - Tissue damage due to inadequate pressure	0
H - Suturing both sides of the vessel	0
I - Vessel rupture	0
J - Inadequate knot technique	88 (98.9)
K - Insufficient vessel preparation	0
L - Inadequate pressure	83 (93.3)
M - Irregular bites	87 (97.8)
N - Excessive suture traction	74 (83.1)
O - Suture rupture due to inadequate pressure	88 (98.9)
P - Lost knots	0
Q - Need to repeat suture	48 (53.9)
R - Inadequate magnification	0
S - Inadequate vessel tension	44 (49.4)
T - Vessel dissection	84 (94.4)
U - Excessively wet surgical field	82 (92.1)
V - Leak (bleeding) through anastomosis	0
X - Inadequate Acland’s patency test	0
Z - Excessive sutures	0
